# Early-life cisplatin exposure induces neuroinflammation and chemotherapy-induced neuropathic pain

**DOI:** 10.1242/dmm.052062

**Published:** 2024-11-27

**Authors:** Marlene Da Vitoria Lobo, Lydia Hardowar, Tameille Valentine, Lucy Tomblin, Charlotte Guest, Dhyana Sharma, Benjamin Dickins, Mark Paul-Clark, Richard Philip Hulse

**Affiliations:** ^1^ Division of Cancer and Stem Cells, School of Medicine, University of Nottingham, Nottingham NG7 2UH, UK; ^2^ Department of Biosciences, School of Science and Technology, Nottingham Trent University, Nottingham NG11 8NS, UK

**Keywords:** Nociceptor, Dorsal root ganglia, Nerve growth factor, Chemotherapy, Cisplatin, Neurodegeneration, TRPV1, Inflammation, Macrophage

## Abstract

Chemotherapy-induced neuropathic pain (CINP) is a common adverse health-related comorbidity that manifests later in life in patients with paediatric cancer. Current analgesia is ineffective, aligning closely with our lack of understanding of CINP. The aim of this study was to investigate how cisplatin induces nerve growth factor (NGF)-mediated neuroinflammation and nociceptor sensitisation. In a rat model of cisplatin-induced survivorship pain, cisplatin induced a neuroinflammatory environment in the dorsal root ganglia (DRG), demonstrated by NGF-positive macrophages infiltrating into the DRG. Cisplatin-treated CD11b- and F4/80-positive macrophages expressed more NGF compared to those treated with vehicle control. Mouse primary DRG sensory neuronal cultures demonstrated enhanced NGF-dependent TRPV1-mediated nociceptor activity after cisplatin treatment. Increased nociceptor activity was also observed when cultured mouse DRG neurons were treated with conditioned medium from cisplatin-activated macrophages. Elevated nociceptor activity was inhibited in a dose-dependent manner by an NGF-neutralising antibody. Intraperitoneal administration of the NGF-neutralising antibody reduced cisplatin-induced mechanical hypersensitivity and aberrant nociceptor intraepidermal nerve fibre density. These findings identify that a monocyte- or macrophage-driven NGF–TrkA pathway is a novel analgesic target for adult survivors of childhood cancer.

## Teaser

**Summary:** In a rodent model of cisplatin-induced pain in adult survivors of paediatric cancer, early-life cisplatin exposure induces neuroinflammation via NGF-mediated nociceptor sensitization, leading to delayed onset of pain.

## INTRODUCTION

Current advances in medical diagnosis and treatment have led to a significant increase in survival rates of patients diagnosed with paediatric cancer (Robison and Hudson, 2014). Unfortunately, owing to the toxic and intensive nature of chemotherapeutic approaches, the patients’ quality of life is severely impacted (Robison and Hudson, 2014). Delayed health complications due to early-life exposure to chemotherapy represents a considerable patient and societal burden, which is complex and unmet (Chang et al., 2022). The most prominent complication that has day-to-day implications for these patients is persistent long-lasting pain, a debilitating condition that is prevalent in up to 59% of childhood cancer survivors post treatment (Alberts et al., 2018; Lu et al., 2011). Pain frequently develops in adolescence and persists into adulthood, well beyond the timeframes of diagnosis and cessation of treatment (Phillips et al., 2015). To date, commonly adopted painkiller approaches include the use of duloxetine (Hershman et al., 2014; Mulvey et al., 2024). However, current clinical analgesic intervention therapy is predominantly ineffective and can cause adverse long-term side effects (Argyriou et al., 2005). This issue, mainly, is promoted by our lack of understanding of how chemotherapy impacts the nociceptor during nervous system maturation.

Platinum-based chemotherapy, including that using cisplatin, is the primary treatment for a wide range of solid tumours that include lung, bladder, testicular and ovarian cancers and hepatoblastoma (Gilchrist and Tanner, 2013; Zsiros et al., 2013). However, treatment is often terminated prematurely due to its adverse side effects. These include chemotherapy-induced neuropathic pain (CINP) in paediatric cancer survivors, which is often reported to be delayed in onset, often manifesting years after the cessation of treatment (Khan et al., 2014; Ness et al., 2013; Phillips et al., 2015). Cisplatin-induced neuropathic pain arises due to treatment-induced neurotoxicity that effects the peripheral somatosensory nervous system (PNS) (Khan et al., 2014), which is an important consideration as the somatosensory nervous system is vulnerable at an early age to cellular stress and damage (Fitzgerald and McKelvey, 2016; Schwaller and Fitzgerald, 2014). Traumatic injury or inflammatory insult during neonatal development causes pain hypersensitivity that is delayed in inception, often presenting during adolescence and persisting throughout adulthood (Fitzgerald, 2005). Previous work using a neonatal rodent model of cisplatin-induced neuropathic pain showed an alteration in the maturation of nociceptors during neuronal development, demonstrated by increased expression of the receptor TrkA (also known as NTRK1) in the dorsal root ganglia (DRG) as well as increased calcitonin gene-related peptide (CGRP)-positive nociceptor sprouting in the skin (Hardowar et al., 2024; Hathway et al., 2018). TrkA signalling is driven by nerve growth factor (NGF), a prominent modulator of nociceptor development and sensitisation (Obreja et al., 2011), and inducer of chronic pain states (Donnerer et al., 1992; Grills and Schuijers, 1998; Longo et al., 2013; Woolf et al., 1994). In addition, a neuroinflammatory component is an important contributor to the delayed manifestation of pain in adulthood, as unsilencing of proinflammatory activity following early-life trauma drives the delayed manifestation of pain (Cobo et al., 2022; McKelvey et al., 2015), with NGF largely derived from the circulatory inflammatory cell types (Minnone et al., 2017). Previous work has outlined that anti-NGF therapy is a potential therapy for chronic pain through suppression of nociceptor sensitisation (Bloom et al., 2011; Sevcik et al., 2005).

This study explores the hypothesis that inflammatory cell type-derived NGF-driven nociceptor sensitisation plays an integral part in the development of delayed pain behavioural phenotype in paediatric cancer survivors. The impact of the cisplatin-induced inflammatory process on nociceptor sensitisation in developing neurons is not fully understood. Here, we used a rodent model of cisplatin-induced pain in adult survivors of childhood cancer (Hathway et al., 2018) to investigate how cisplatin-induced neuroinflammation induces nociceptor sensitisation in an NGF-dependent manner.

## RESULTS

**Fig. 1.**
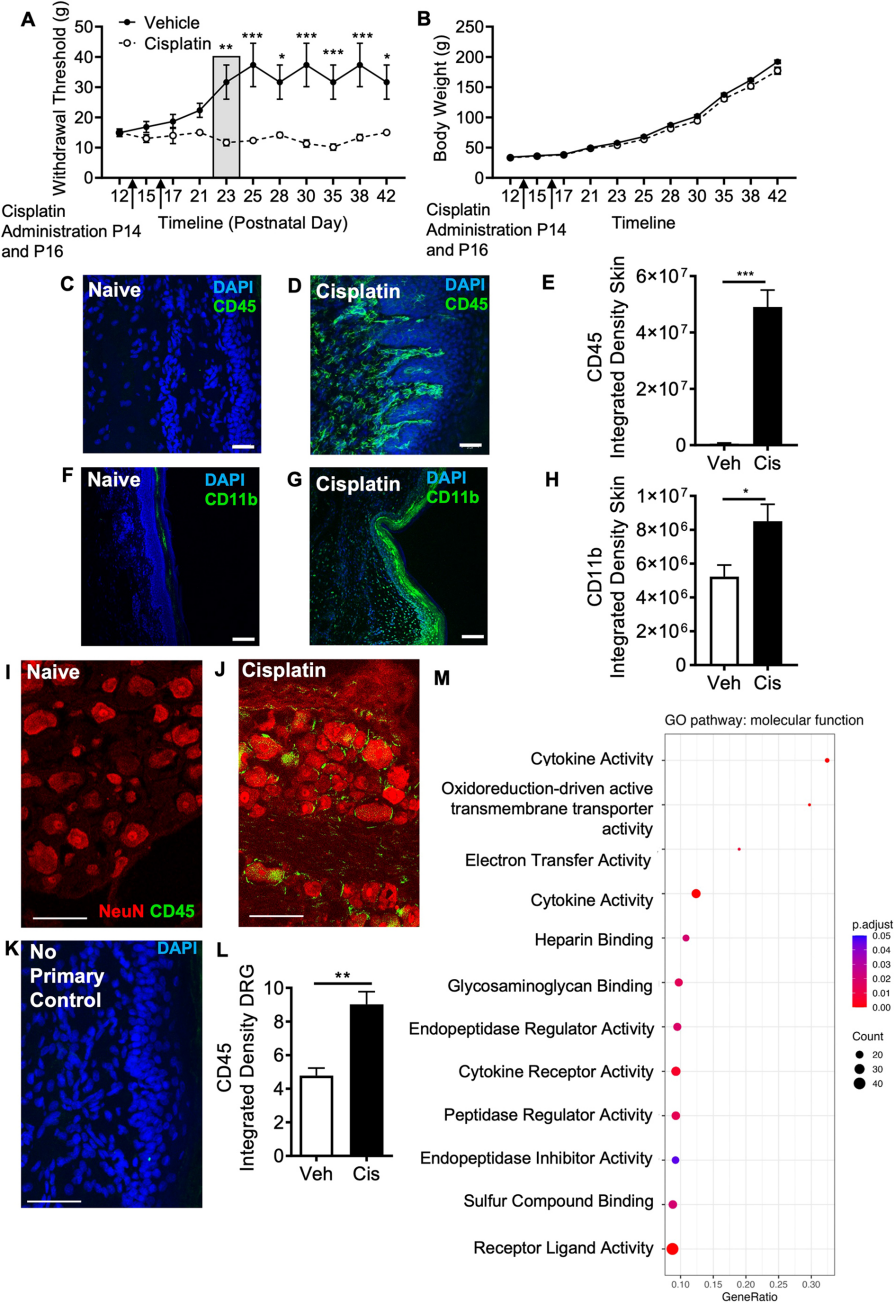
**Early-life exposure to cisplatin induces mechanical allodynia and DRG macrophage infiltration.** (A) Neonatal exposure to cisplatin [0.3 mg/kg intraperitoneal injection delivered on two occasions; postnatal (P) days 14 and 16; timepoints indicated by arrows] led to a delayed (presenting at P23) but pronounced mechanical allodynia compared to that in age-matched sham control (vehicle)-treated rats. (B) There were no differences in body weight between vehicle- and cisplatin-treated rats (two-way ANOVA with Bonferroni’s post hoc test, *n*=6 animals per group). (C-M) Early-life exposure of cisplatin led to an increase in proinflammatory environment in the peripheral sensory nervous system – the dorsal root ganglia (DRG) and plantar skin of the hindpaw. (C-H) Representative images of plantar skin sections from age-matched vehicle-treated controls and cisplatin-treated rats stained for CD45 (C,D) and CD11b (F,G) are shown. There was an increase POS=--10ptin CD45-positive cell types (E) in the plantar skin of the hindpaw in cisplatin-treated rats versus those in age-matched controls (two-tailed unpaired *t*-test, *n*=6 animals per group). Similarly, there was an increase in CD11b-positive cells (H) in the plantar skin of the hindpaw in cisplatin-treated rats versus those in age-matched controls (two-tailed unpaired *t*-test, *n*=6 animals per group). (I-K) Compared to the sham vehicle-treated DRG (I), cisplatin-treated DRG (J) demonstrated increased CD45 immunoreactivity. Representative images of controls with no primary antibody staining are also shown (K). (L) In the DRG (lumbar 5), there was an increase in CD45-positive cells in cisplatin-treated rats compared to that in age-matched sham controls (two-tailed unpaired *t*-test). (M) Whole-lumbar-DRG RNA-sequencing analysis highlighted that cisplatin induced the expression of genes enriched in Gene Ontology Molecular Function terms indicative of an inflammatory neural environment. Bars show mean±s.e.m. Scale bars: 25 μm (C,D,I,J); 50 μm (F,G). *P<0.05; **P<0.01; ***P<0.001.

Early-life administration of cisplatin in neonatal rats [postnatal day (P)14 and P16] induced a delayed but pronounced mechanical allodynia (Fig. 1A), with no observed alterations in body weight (Fig. 1B). In the plantar skin (Fig. 1C,D), cisplatin induced an increased infiltration and accumulation of CD45 (also known as PTPRC)-positive monocytes/macrophages (Fig. 1E) versus those in age-matched vehicle-treated controls. Similarly, in comparison from the plantar skin of age-matched vehicle-treated controls, there was an increase in CD11b (also known as ITGAM)-positive cell number in the plantar skin from cisplatin-treated rats (Fig. 1F-H). Additionally, cisplatin also induced infiltration of CD45-positive monocytes/macrophages in the lumbar DRG (Fig. 1I,J,L) compared to that in age-matched vehicle-treated controls (a representative control image without primary antibody staining is shown in Fig. 1K). Furthermore, following transcriptomic analysis at the P23 timepoint (associated with the development of CINP), there was an increase in the expression of genes associated with the proinflammatory environment in lumbar DRGs following early-life treatment with cisplatin, compared to that in age-matched vehicle-treated controls (Fig. S1A-E). In rats administered cisplatin during the second week of life, a significant inflammatory profile in the DRG was observed using gene enrichment analysis (Fig. 1M,N; Fig. S1A; 220 differentially expressed genes – 143 downregulated and 77 upregulated). Overall, DRG and plantar skin at a timepoint accompanying presentation of pain demonstrated infiltration and accumulation of CD45 and CD11b cell types in the PNS.

**Fig. 2.**
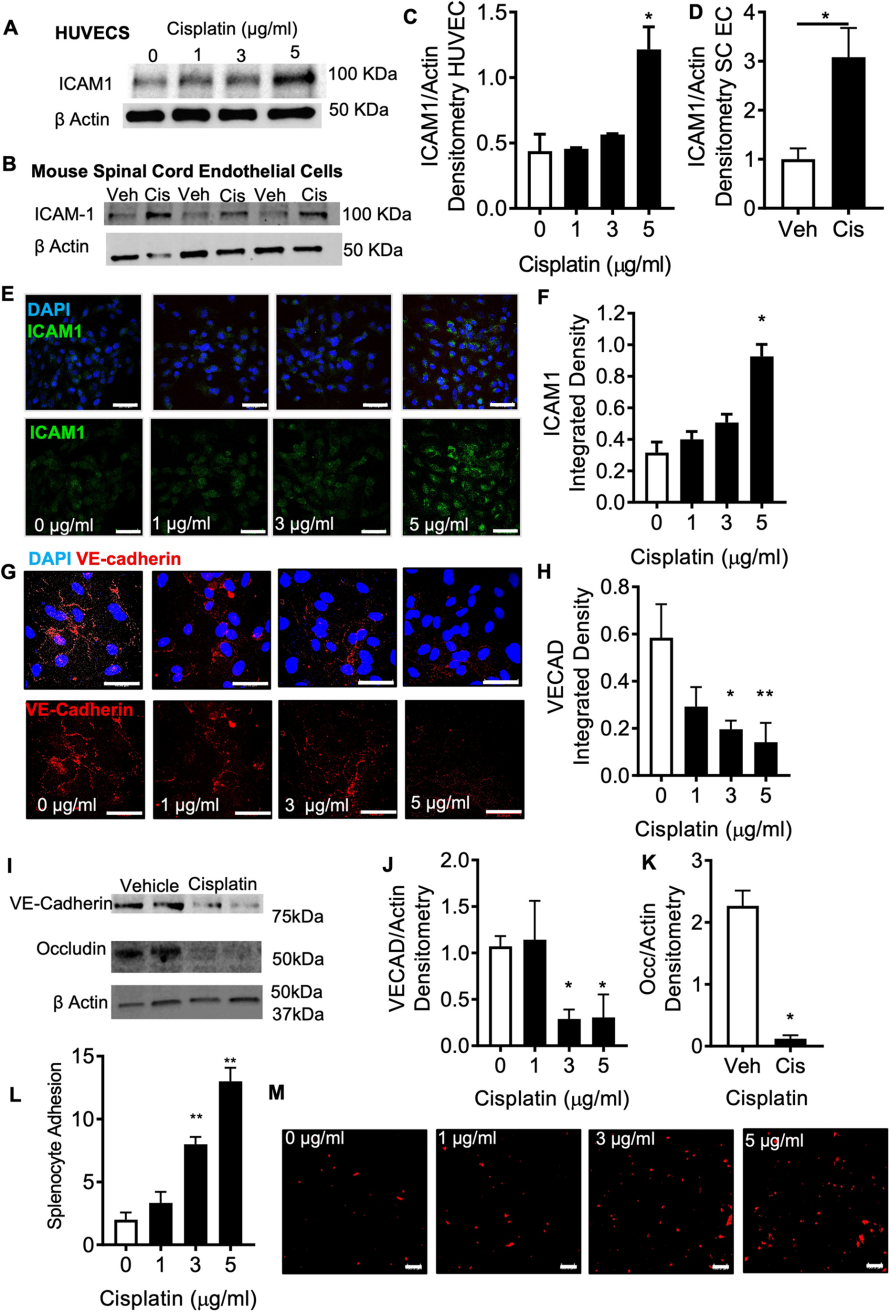
**Cisplatin induced an inflammatory environment in the DRG.** (A-D) Cisplatin induced increases in ICAM1 expression in HUVECs (*n*=4 per group) (A,C) and mouse spinal cord endothelial cells (SC ECs) (*n*=3 per group) (B,D) as measured by densitometry analysis. (E,F) Similarly, immunocytochemistry analysis (E) showed that ICAM1 expression was increased in HUVECS 24 h post cisplatin treatment, with increases occurring in a dose-dependent manner (cisplatin concentrations of 0, 1, 3 or 5 μg/ml; *n*=4 per group (F). (G,H) HUVECs treated with cisplatin showed reductions in the endothelial tight junctional marker VE-cadherin (VECAD). (I-K) Protein lysates extracted from HUVECs after 24 h treatment with 0, 1, 3 or 5 μg/ml of cisplatin demonstrated reductions in VE-cadherin (I,J) and occludin (I,K) expression (*n*=4 per group). (L,M) Immunocytochemistry of HUVECs treated for 24 h with either vehicle or cisplatin demonstrated increased adherence of mouse splenocytes fluorescently labelled with Vybrant Dil with increasing concentrations of cisplatin (*n*=3 per group). Bars show mean±s.e.m. (C,F,H,J,L) One-way ANOVA with Bonferroni test; (D,K) two-tailed unpaired *t*-test. Scale bars: 50 μm. *P<0.05; **P<0.01.

Cisplatin treatment was associated with alterations in endothelial cell function in relation to permeability and cell adhesion (Fig. 2). Increased inflammatory cell–endothelial cell adherence was associated with elevations in ICAM1 expression in cisplatin-treated endothelial cells [human umbilical vein endothelial cells (HUVECs) and mouse spinal cord endothelial cells] (Fig. 2A-F). Additionally, cisplatin induced loss of tight junctional proteins [VE-cadherin (also known as CDH5; Fig. 2G-J) and occludin (OCLN; Fig. 2I,K)], which can promote increased capillary leakiness and cell trafficking. Here, we demonstrate that there is enhanced inflammatory cell adherence promoting inflammation into the DRG. This was shown in cisplatin-treated HUVECs having increased adherence of mouse splenocytes fluorescently labelled with Vybrant Dil compared to vehicle-treated cells (Fig. 2L,M).

**Fig. 3.**
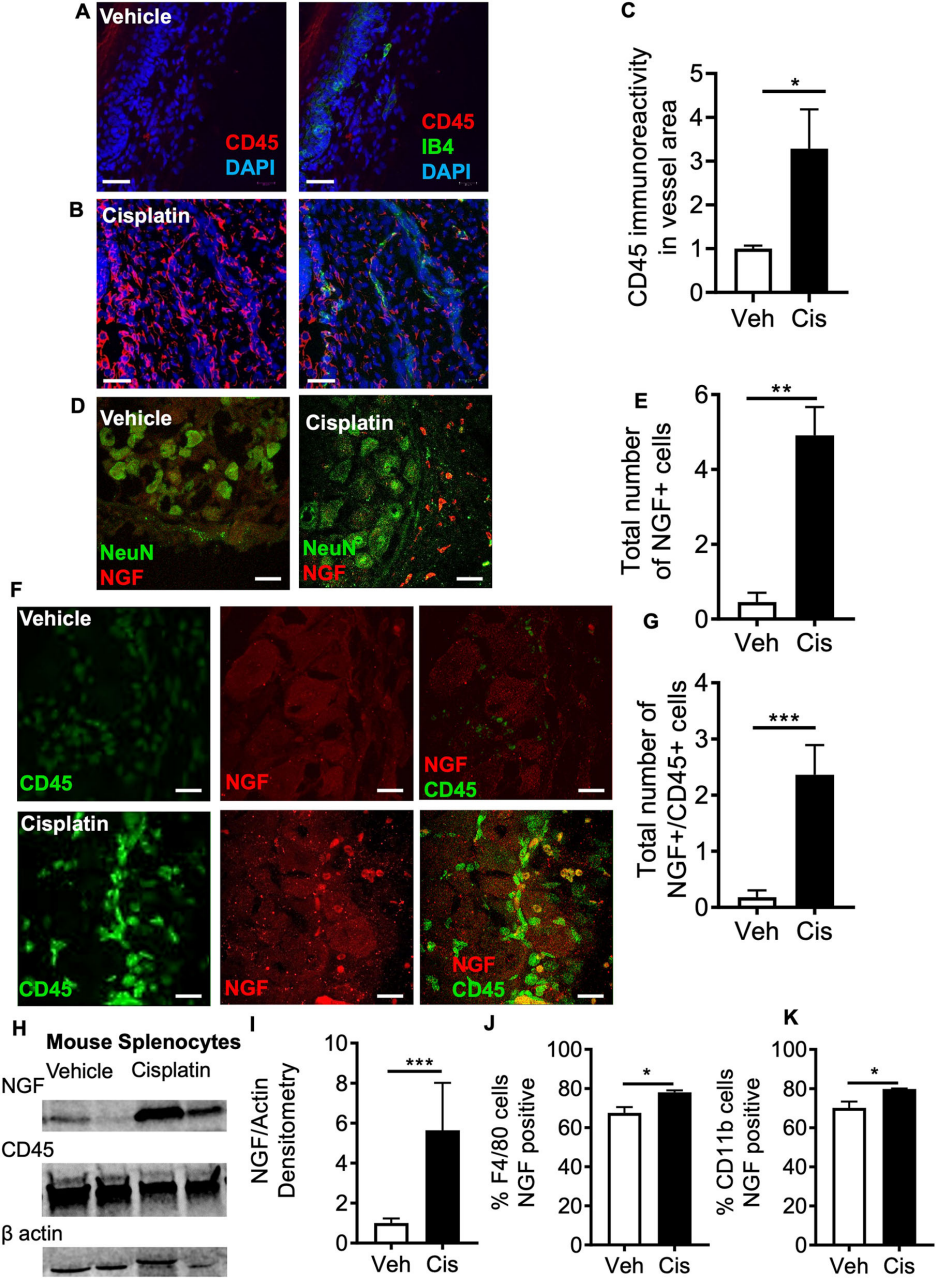
**Cisplatin induced accumulation of NGF-positive monocytes/macrophages in the DRG.** (A-C) In rats treated with cisplatin early in life, there was an increased accumulation of CD45-positive cells in the plantar skin that were associated with IB4-positive microvessels, compared to that seen in age-matched control animals (*n*=4 per group). (D,E) In rats treated with cisplatin early in life, there was an increased number of NGF-positive cells in the DRG. (F,G) Furthermore, there was an increase in number of NGF- and CD45-positive cells in the DRG of cisplatin-treated rats versus sham vehicle-treated control rats. (H-K) Western blotting (H,I) and flow cytometry (J,K) analyses of isolated mouse splenocytes treated with either vehicle or cisplatin for 24 h showed increased expression of NGF following cisplatin treatment (H,I) and an increased number of F4/80 (J) and CD11b (K)-positive cells expressing NGF (*n*=4 per group). Bars show mean±s.e.m. (C,E,G,I-K) Two-tailed unpaired *t*-test. All scale bars: 25 μm. *P<0.05; **P<0.01; ***P<0.001.

There was an increase in CD45 inflammatory cell accumulation in the plantar surface of the hindpaw of cisplatin-treated rats, which was associated with the microvessel endothelium (labelled with isolectin B4 or IB4), compared to that in age-matched vehicle-treated controls (Fig. 3A-C), indicative of enhanced monocyte/macrophage adherence and infiltration. In the lumbar DRG extracted from cisplatin-treated rats, there was increased accumulation of NGF-positive cells (Fig. 3D,E), versus that in age-matched controls. Increased expression of NGF following cisplatin treatment was also observed in our transcriptomic analyses (Fig. S1A). This accumulation of NGF in the DRG aligned with an increase in infiltrating CD45-positive monocytes/macrophages, with CD45-positive monocytes/macrophages expressing NGF (Fig. 3F,G). In isolated mouse splenocytes, cisplatin treatment induced increased expression of NGF compared to that in vehicle-treated splenocytes, as seen by western blotting (Fig. 3H,I), and also increased the number of F4/80 (also known as ADGRE1; Fig. 3J) and CD11b (Fig. 3K)-positive cells that expressed NGF post cisplatin treatment (Fig. S2), as seen by flow cytometry.

**Fig. 4.**
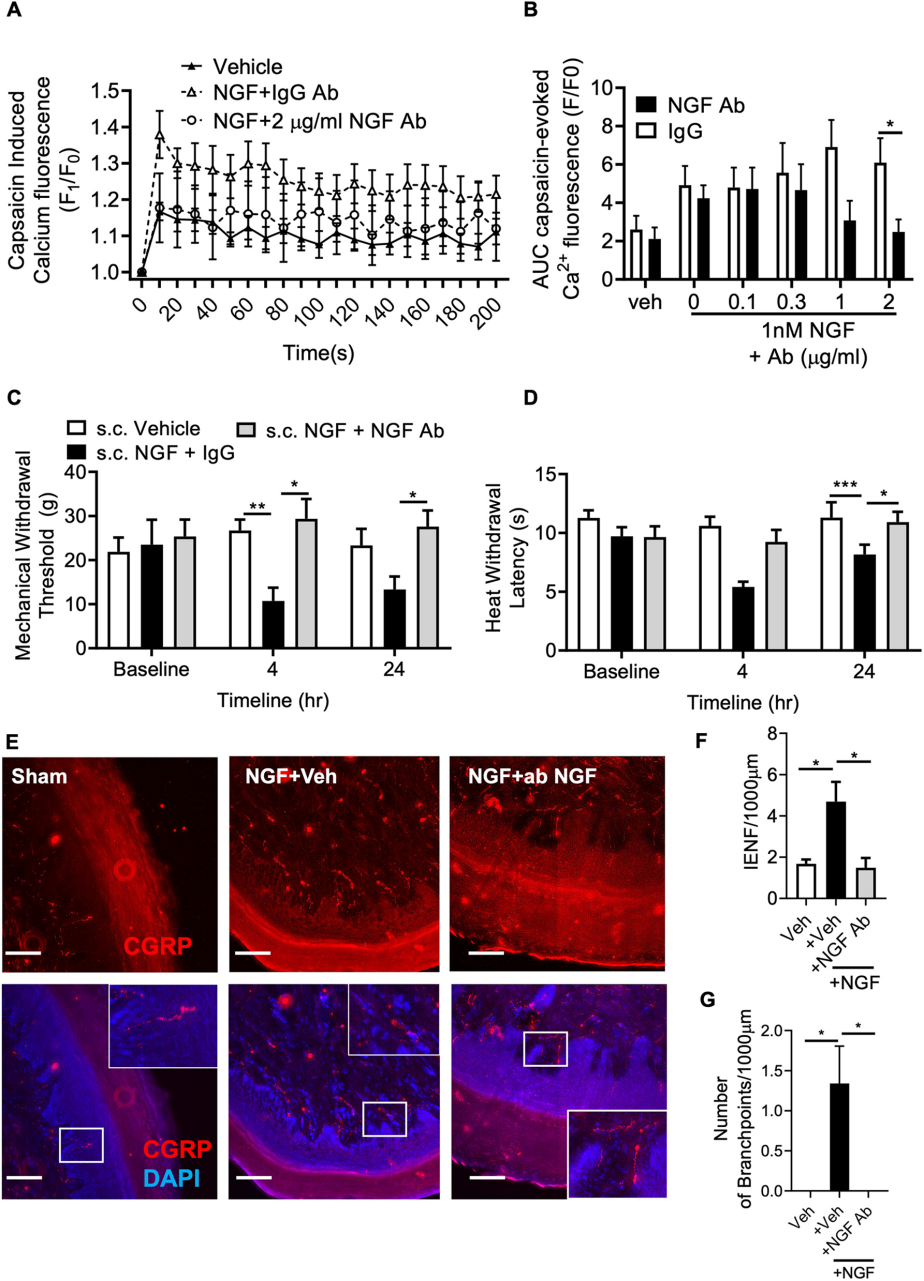
**Suppression of NGF by an NGF-neutralising antibody induced nociceptor sensitisation and aberrant growth.** (A,B) In isolated mouse DRG primary sensory neuronal cell cultures, NGF treatment induced an increase in capsaicin-mediated intracellular calcium influx, which was suppressed in a concentration-dependent manner with increasing concentrations of NGF-neutralising antibody (*n*=16 for PBS and NGF+0 μg/ml antibody treatment groups, *n*=14 for vehicle treatment, *n*=10 for NGF+0.1, 0.3, 1 and 2 μg/ml antibody treatment groups; one-way ANOVA with Bonferroni test). AUC, area under the curve. (C-G) Compared to NGF treatment alone, subcutaneous (s.c.) injection in the hindpaw plantar surface of rats with NGF plus the NGF-neutralising antibody prevented NGF-induced mechanical (C) and heat (D) hyperalgesia (one-way ANOVA with Bonferroni test, *n*=6 per group), alongside reductions in NGF-induced aberrant growth of CGRP-positive nociceptor intraepidermal nerve fibres (IENFs) (E,F) and an increase in the number of branchpoints (G) in the ipsilateral plantar skin (one-way ANOVA with Bonferroni test, *n*=6 per group). Bars show mean±s.e.m. Scale bars: 100 μm. *P<0.05; **P<0.01; ***P<0.001.

Increasing concentrations of capsaicin applied to isolated mouse DRG sensory neurons induces increased intracellular calcium influx (Hardowar et al., 2024). Here, NGF treatment led to increased capsaicin-induced intracellular calcium influx (Fig. 4A,B) in mouse DRG nociceptors. Furthermore, cotreatment with NGF and an NGF-neutralising antibody inhibited NGF-induced DRG nociceptor sensitisation, whereas cotreatment with NGF and an IgG control antibody did not suppress NGF-induced nociceptor sensitisation (Fig. 4A,B; Fig. S3). Subcutaneous administration in the hindpaw plantar surface of rats with NGF and the NGF-neutralising antibody inhibited NGF-induced mechanical (Fig. 4C) and heat (Fig. 4D) hypersensitivity in the ipsilateral hindpaw. Similarly, intraperitoneal delivery of the NGF-neutralising antibody also prevented NGF-induced pain (Fig. S4). Subcutaneous administration of NGF into the plantar surface of the hindpaw led to increased numbers of CGRP-positive intraepidermal nerve fibres (IENFs) and branchpoints in the ipsilateral plantar skin (Fig. 4E-G, representative images of CGRP-positive IENFs are shown in Fig. 4E) versus those in the vehicle-treated group. NGF administered in conjunction with the NGF-neutralising antibody inhibited the NGF-induced increase in the number of CGRP-positive IENFs and branchpoints (Fig. 4E-G).

**Fig. 5.**
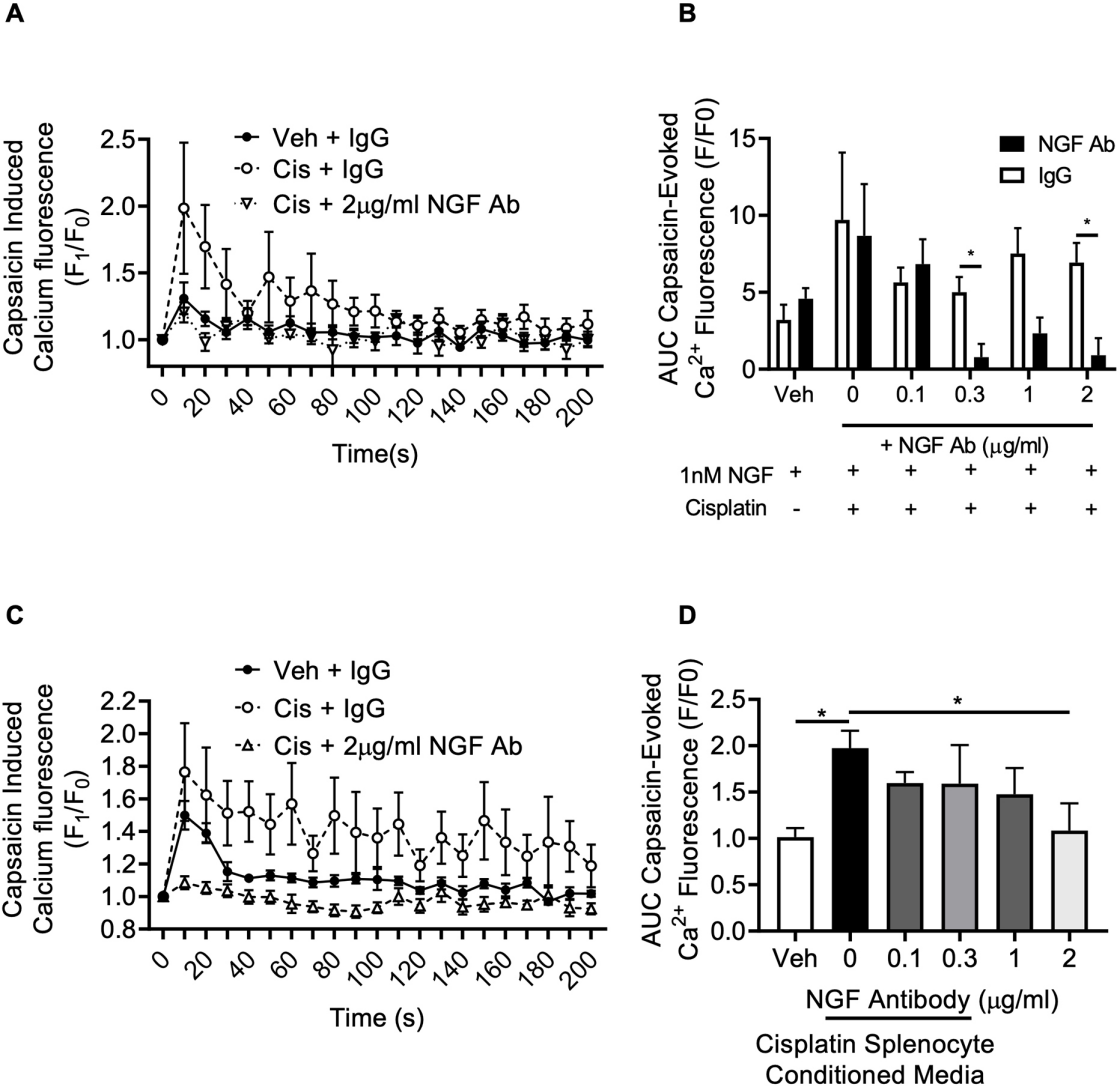
**Cisplatin-induced proinflammatory-mediated nociceptor sensitisation is NGF dependent.** (A,B) Treatment of mouse DRG primary cell cultures with 5 μg/ml cisplatin 1 week prior to NGF treatment increased TRPV1 activity compared to that following vehicle treatment. Treatment with the NGF-neutralising antibody diminished capsaicin-induced TRPV1-mediated nociceptor activity (one-way ANOVA with Bonferroni test, *n*=12 per group). (C,D) 24 h exposure of mouse DRG primary cell cultures to conditioned medium from cisplatin-treated mouse splenocytes led to increased capsaicin-evoked DRG nociceptor activity compared to that in cultures exposed to vehicle-treated conditioned medium from mouse splenocytes, and this increase was diminished with increasing concentrations of the NGF-neutralising antibody (one-way ANOVA with Bonferroni test, *n*=9 per group). Bars show mean±s.e.m. AUC, area under the curve. *P<0.05.

**Fig. 6.**
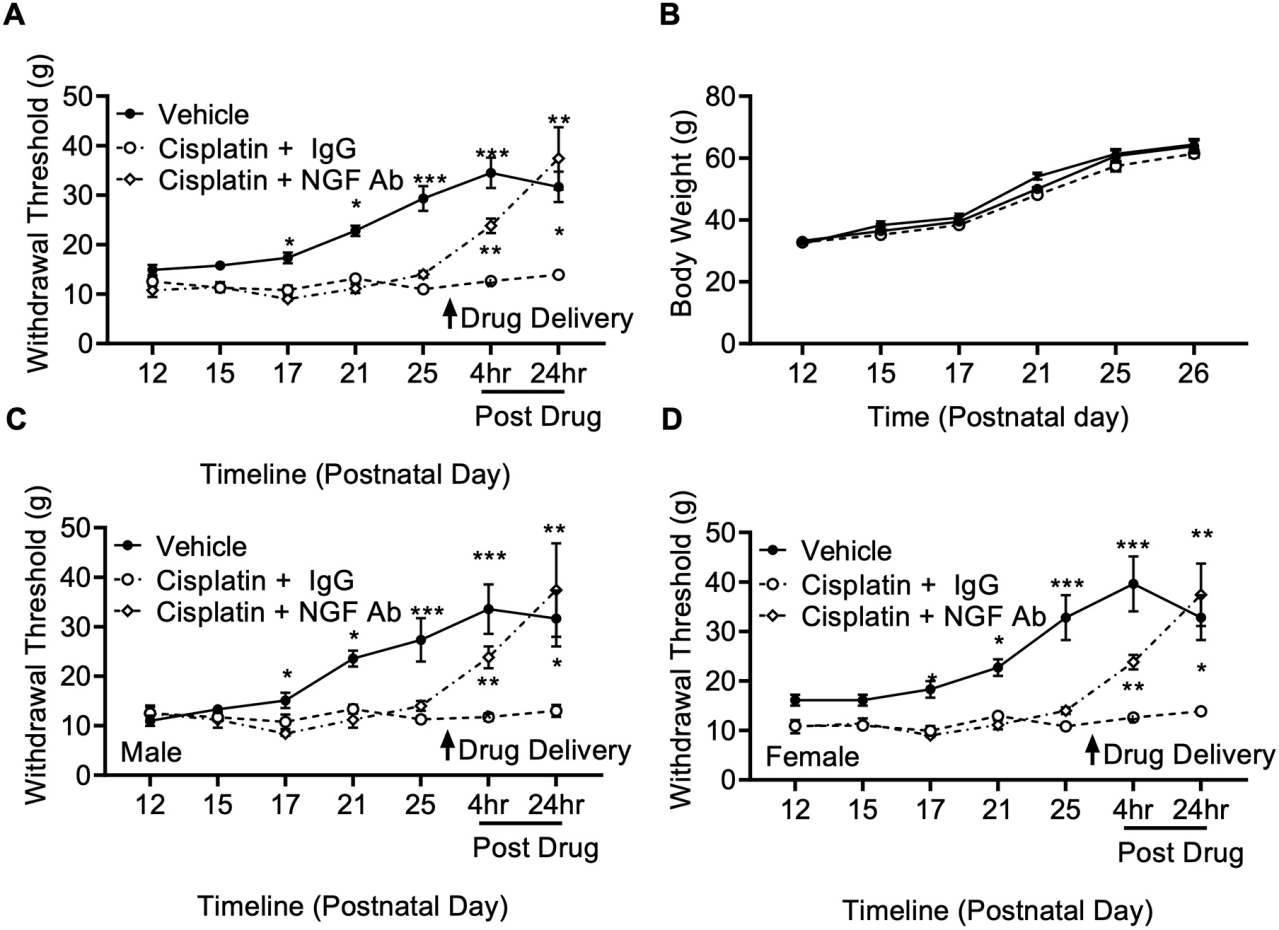
**NGF-mediated cisplatin-induced survivorship pain.** (A) In a rat model of early-life exposure to cisplatin, which induced chronic pain, intraperitoneal injection of the NGF-neutralising antibody prevented mechanical allodynia versus that seen in the group treated with cisplatin and an IgG control antibody. (B) Body weight did not differ between each experimental group. (C,D) In addition, compared to the cisplatin+IgG group, intraperitoneal injection of the NGF-neutralising antibody prevented mechanical allodynia in cisplatin-treated male (C) and female (D) rats. Bars show mean±s.e.m. *P<0.05, **P<0.01, ***P<0.001 (two-way ANOVA with Bonferroni test; *n*=9 vehicle-treated animals, 10 cisplatin+IgG-treated animals, 5 cisplatin+NGF antibody-treated animals per group).

**Fig. 7.**
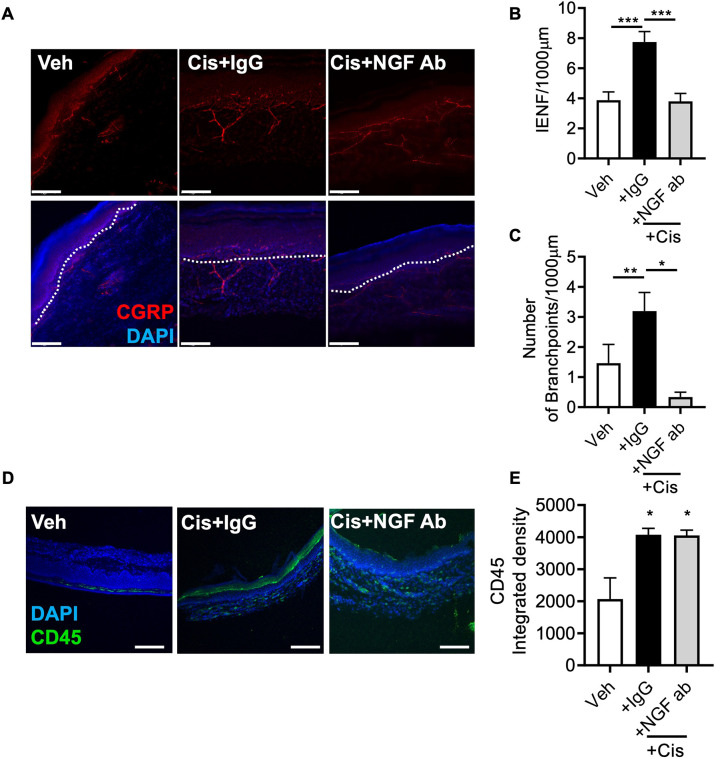
**Cisplatin-induced aberrant IENF growth is NGF dependent.** (A-C) Early-life exposure of rats to cisplatin led to increased CGRP-positive nociceptor intraepidermal nerve fibres (IENFs) in the plantar skin versus those in sham vehicle-treated CGRP-positive nociceptors (A,B) and increased IENF branch number (C). In addition, treatment with the NGF-neutralising antibody prevented cisplatin-induced aberrant growth and branch number in CGRP-positive IENFs. Dotted lines in A represent dermal–epidermal border. (D) Cisplatin treatment induced a proinflammatory environment (as seen by the increased number of CD45-positive cells) in the plantar skin versus the sham vehicle-treated group. (E) The proinflammatory state (CD45-positive cell infiltration) within the plantar skin remained unchanged between the cisplatin- and cisplatin+NGF-neutralising antibody-treated groups. Bars show mean±s.e.m. Scale bars: 100 μm. *P<0.05; **P<0.01; ***P<0.001 (one-way ANOVA with Bonferroni test, *n*=5 per group).

Mouse DRG primary cell cultures were treated with cisplatin for 24 h, followed by a 7-day washout period. NGF-induced TRPV1 sensitisation was exacerbated by cisplatin treatment, and treatment in conjunction with the NGF-neutralising antibody diminished NGF-induced capsaicin responses compared to those seen upon cotreatment with the IgG control antibody (Fig. 5A,B; Fig. S5). Isolated mouse splenocytes were treated with either vehicle or cisplatin. Conditioned media were collected 24 h later and applied to DRG primary cell cultures. Capsaicin-induced intracellular calcium influx was increased in response to cisplatin-treated conditioned medium (Fig. 5C,D). Furthermore, with increasing concentrations of the NGF-neutralising antibody, cisplatin-treated conditioned medium-induced nociceptor sensitisation was inhibited (Fig. 5C,D). In a rat model of early-life cisplatin-induced chronic pain, mechanical allodynia was prevented following intraperitoneal injection of the NGF-neutralising antibody (Fig. 6A), with body weight remaining similar between all experimental groups (Fig. 6B). In addition, intraperitoneal injection of the NGF-neutralising antibody prevented cisplatin-induced mechanical allodynia in male (Fig. 6C) and female (Fig. 6D) rats. Furthermore, cisplatin-induced increases in CGRP-positive nociceptor IENFs in the plantar skin were prevented in the NGF-neutralising antibody treated group (Fig. 7A-C). In addition, cisplatin-induced accumulation of CD45-positive cells in the plantar hindpaw skin was not altered by administration of the NGF-neutralising antibody (Fig. 7D,E).

## DISCUSSION

In this study, we demonstrate that early-life treatment with cisplatin causes neuroinflammation, which is a major contributor to delayed and persistent neuropathic pain states that occur in adult survivors of childhood cancer. Results were comparable between male and female rats. Cisplatin-induced pain and nociceptor sensitisation was driven by infiltrating M1-like macrophages into the PNS in an NGF-dependent manner. This investigation outlines an inflammatory mechanism that induces the onset of pain in adult survivors of childhood cancer.

### Neuroinflammation-induced childhood cancer survivorship pain

Current clinical studies highlight the development of a lasting pain in adult individuals who were treated during infancy for paediatric cancer. However, the ongoing underlying mechanisms that cause this sensory neuropathology have not been elucidated. Recently, several rodent models have been developed to enable exploration of the aetiology of this complication (Hathway et al., 2018; Schappacher et al., 2019). These preclinical studies clearly demonstrate a disturbance in the maturation of the nociceptor following exposure to chemotherapy early in life, and, importantly, pain hypersensitivity developing during adolescence, a timepoint comparable to those observed in clinical studies (Alberts et al., 2018). It is widely regarded that cisplatin-induced neuropathic pain is dependent upon sensory neurotoxicity that occurs due to the accumulation of platinum-based agents in the PNS (McDonald et al., 2005; Ta et al., 2006), inducing nociceptor sensitisation (Ferdousi et al., 2015). This is supported by clinical evidence indicating that cisplatin can alter sensory neural activity in survivors of childhood cancer (Kandula et al., 2020). However, in relation to early-life exposure to cisplatin, how pain manifests and the underlying mechanisms still remain unclear. Current understanding of developmental sensory neurobiology indicates that the delay in the presentation of neuropathic pain phenotypes until adolescence following early-life exposure to a ‘neural stressor’ aligns with the induction of proinflammatory processes (McKelvey et al., 2015). Exposure of the developing immature PNS to a traumatic/toxic insult initiates maladaptation within the developmental trajectory of the nociceptor and surrounding environment to drive a delayed but lasting chronic pain phenotype in humans (Khan et al., 2014; Ness et al., 2013) and rodents (Fitzgerald and McKelvey, 2016; McKelvey et al., 2015). Part of this is attributed to the unsilencing of the immune system during adolescence, which, during neural development, is normally anti-inflammatory in nature, preventing nociceptor activation (McKelvey et al., 2015). It is important to note that organism development differs suitably between rodents and humans. This is exemplified by rodents transitioning from infants to sexual maturity at a significantly faster rate than humans. However, the underpinning neural mechanisms attributable to infant and adolescent pain are comparable between organisms (Fitzgerald, 2005). It has previously been shown that cisplatin drives inflammation in a multitude of differing physiological systems, for instance, in cisplatin-induced nephropathy (Liang et al., 2016), where macrophage infiltration is associated with pathophysiological progression. Early-life exposure to cisplatin promotes, during adolescence, alterations in the vascular–immune interactions, demonstrated by increased PNS capillary endothelium permeability that aligns with enhanced macrophage adhesion, trafficking and penetration. This is similar to what has been reported in alternative rodent pain model systems. Following traumatic nerve injury early in life (7-10 days), inflammatory activation does not develop in the somatosensory nervous system until post weaning (∼21 days) (Vega-Avelaira et al., 2012, 2009). This is in contrast to that observed in adults, in which tissue inflammation is induced within less than 7 days. Importantly, this is accompanied by pain hypersensitivity. This is significant as pain hypersensitivity following a traumatic nerve injury does not develop until a delayed proinflammatory state is established within the somatosensory nervous system (McKelvey et al., 2015). These findings align with our observations of a delayed cisplatin-induced proinflammatory state that ensues during adolescence, when neuropathic pain is presented. Furthermore, cisplatin has been shown to drive M1 macrophage polarisation with the accompanying expression of proinflammatory mediators in adult chemotherapy-induced peripheral neuropathy (CIPN) (Starobova et al., 2020; Valentine et al., 2022). This is particularly pertinent as cisplatin-induced survivorship pain is associated with an increased infiltration of CD45 immunoreactivity, which is restricted to the DRG, depicting neuroinflammatory activation. Furthermore, infiltrating macrophages initiate a DRG microenvironment that is abundant with nociceptor-sensitising agents or inflammatory mediators (Guimarães et al., 2023). Exposing developing nociceptors to the secretome of cisplatin-treated macrophages led to nociceptor sensitisation, indicating that cisplatin activates macrophages to initiate nociceptor sensitisation and delayed pain in rodents via an inflammatory process.

### NGF-dependent induction of childhood cancer survivorship pain

Previously, early exposure to cisplatin led to alterations in the maturation of the developing nociceptors (Hathway et al., 2018). Cisplatin drives neural stress and damage exemplified by the induction of neurodegenerative processes that include diminished IENF innervation (Vencappa et al., 2015), with cisplatin-induced sensory neurotoxicity widely recognised in adult model systems (Ta et al., 2009; Vencappa et al., 2015). This sensory neurodegeneration has also been identified in rodent models of chemotherapy-induced childhood cancer pain (Schappacher et al., 2017). However, our previous work has demonstrated that, following a ‘washout’ period, a delayed cisplatin-induced survivorship pain develops in association with aberrant nociceptor growth following initial neurotoxicity and degeneration of nociceptor nerve terminals (Hathway et al., 2018). This regenerative capacity of nociceptors is supported by recent evidence that, following an initial lesion of the nociceptor, there is a pronounced recovery of nociceptor sensory afferent terminals (Gangadharan et al., 2022). However, this nociceptor recovery is accompanied by neuropathic pain phenotypes that were dependent upon CGRP nociceptors. Exaggerated abnormal IENF growth, particularly with CGRP-labelled nociceptors, has been widely established to align with the onset of chronic pain states. This has been associated in times of tissue inflammation such as during cancer (Bloom et al., 2011; Mantyh et al., 2010) and arthritic pain (Jimenez-Andrade and Mantyh, 2012). Cisplatin-induced aberrant growth of CGRP-positive nociceptor nerve terminals is dependent upon the proinflammatory environment in the PNS that accumulates during adolescence, with this being associated with elevated local levels of NGF. Inflammation and the local inflamed tissue environments promote hyperalgesia and nociceptor sensitisation through the release of several inflammatory mediators or nociceptor sensitisers (Pinho-Ribeiro et al., 2017). Cisplatin has been found to activate the immune system and plays a significant role in the mechanisms behind CIPN manifestation. Cisplatin-induced sensory neurotoxicity in the DRG leads to the recruitment of immune cells to the site of neurotoxicity and the release of proinflammatory mediators, such as chemokines and cytokines. As presented here, other studies have found that elevated levels of Il1β, Il6, TNFα and NGF are part of the inflammatory response associated with CIPN (Valentine et al., 2022). In this study, we have only focussed upon NGF contributions; however, as mentioned, other inflammatory mediators may also have a significant contribution to cisplatin-induced pain and this warrants further exploration. In addition, it must be noted that fundamental to the efficient function of the PNS are the supporting glial and inflammatory cell types that support metabolic demands to accommodate functional outputs to support sensory neuronal function (van der Vlist et al., 2022). Additionally, neurosurvival or neuroregenerative capabilities are promoted through engaging with inflammatory mediators such as NGF (Hayakawa et al., 1998), and this NGF-dependent neuroregenerative capacity has previously been shown to prevent pain (Aloe et al., 2012; Ramer et al., 2000).

As previously outlined, the neuroinflammatory process is fundamental to the development of chronic pain in adults as well as in children. There are numerous factors that influence the developmental trajectory of nociceptors and hardwiring of specialised nerve endings in the skin, with NGF as a primary modulator. NGF coordinates and establishes IENFs during development, with fluctuations in elevated NGF expression occurring during different stages. During the embryonic phase, sensory afferent terminals begin to innervate the epidermis/dermis, with IENF hardwiring establishing at 3 weeks of age aligning with peaks in NGF expression (Constantinou et al., 1994). However, surgical intervention (Constantinou et al., 1994) and induction of local tissue inflammation (Bull et al., 1998; Woolf et al., 1994) within the first week of life induces elevations in NGF expression. This is associated with neurogenesis of sensory afferent terminals depicted through elevated levels of neuritogenesis-inducing proteins such as growth-associated protein 43 (GAP43), increasing nociceptor skin innervation (Leslie et al., 1995). Here, we show that increased NGF abundance in the PNS post cisplatin treatment is associated with infiltrating macrophages and presentation of neuropathic pain. The upregulation of NGF is thought to stimulate the hyperexcitability and sensitisation of peripheral nociceptive neurons that cause the perceived pain sensation in patients with CIPN. The DRG are particularly vulnerable to inflammatory damage and accumulation of platinum-based chemotherapy due to a highly permeable protective barrier (Jimenez-Andrade et al., 2008). There have been extensive studies regarding peripheral sensitisation by varying inflammatory mediators in response to injury, including the role of NGF–TrkA signalling in the pathophysiology of peripheral sensory neuropathy. NGF is associated with nociceptor-mediated hyperalgesia and long-term nociceptor sensitisation, through hyperalgesic priming (Ferrari et al., 2010) and unmasking of silent nociceptors (Prato et al., 2017) – neural processes that lead to long-term maladaptation of the nociceptor. This indicates that alterations in NGF handling is integral to the manifestation of neuropathic pain states. As NGF–TrkA signalling has been established as a key mediator in the development of neuropathic pain, inhibition of this pathway appears as an attractive target as a novel analgesic therapy. Previous work has demonstrated that early-life treatment with cisplatin alters the maturation of nociceptors, resulting in an increase in TrkA-positive neurons (Hardowar et al., 2024; Hathway et al., 2018). Importantly, patients treated with cisplatin have been reported to have elevated serum NGF concentrations (Velasco et al., 2017), highlighting the NGF–TrkA signalling axis as a potential mediator of cisplatin-induced neuropathic pain. Here, we also demonstrate that suppression of NGF-dependent signalling prevents cisplatin-induced nociceptor sensitisation and survivorship pain.

Our findings establish NGF-dependent modulation of cisplatin-induced DRG nociceptor sensitisation and consequent development of chronic pain states. The results from this study indicate that cisplatin treatment exacerbates NGF-mediated nociceptor sensitisation and initiates chemotherapy-induced pain in adult survivors of childhood cancer. Furthermore, these findings demonstrate the clinical relevance of developing an antibody-based novel analgesic to inhibit NGF-mediated nociceptor signalling, to treat the painful symptoms of chemotherapy induced peripheral neuropathy in childhood survivors of cancer.

## MATERIALS AND METHODS

### Ethical approval and animals used

All experimental studies involving animals were performed following consultation with the local Animal Welfare and Ethics Review Board at Nottingham Trent University and in accordance with the UK Home Office Animals (Scientific procedures) Act 1986 and ARRIVE guidelines. Animals had *ad libitum* access to standard chow and were housed under 12 h:12 h light:dark conditions.

### Induction of cisplatin-induced neuropathic pain and administration of pharmacological agents

Neonatal Wistar rats were administered either vehicle (phosphate-buffered saline; PBS) or cisplatin (Sigma-Aldrich; 0.3 mg/kg) via intraperitoneal injection delivered on two occasions, P14 and P16, and were randomly allocated to experimental groups. All rats were weaned no later than 22 days and group housed according to gender. Male and female Wistar rats were used in all outlined studies presented in this article, including nociceptive behavioural assays and immunofluorescence analyses, with animal number outlined in the figure legends. Body weight was monitored regularly throughout the study, with no observed reductions in body weight identified. Cisplatin did not cause any compromise to animal health and no rat was culled or removed from the study. All data from all studies have been included in this study.

### Nociceptive behaviour

All rats were habituated to the testing environment prior to nociceptive behavioural experimentation (Da Vitoria Lobo et al., 2022; Drake et al., 2021). Mechanical withdrawal thresholds were acquired following application of von Frey (vF) hairs to the plantar surface of the hindpaw. Withdrawal thresholds were calculated following application of differing vF filaments of increasing force, with each vF filament applied a total of five times to the plantar surface of the hindpaw. Force response withdrawal curves were generated, and mechanical withdrawal thresholds were determined as the mechanically applied force to elicit 50% of nociceptive withdrawals. The Hargreaves test was performed to determine heat nociceptive withdrawal latency (Hargreaves et al., 1988). A radiant heat source was applied to the plantar surface of the hindpaw. The duration (latency) between onset of stimulus to the rats withdrawing their paws was recorded as the withdrawal latency. This was measured three times, and the mean latency was calculated for both hindpaws.

### Drug delivery

NGF 2.5S (NGF, Alomone) was administered at a concentration of 1 μM to rats via a 20 μl subcutaneous injection under recovery anaesthesia (∼2% isoflurane in O_2_). In some instances, the NGF-neutralising antibody (goat anti-NGF, Sigma-Aldrich, N8773) was administered via intraperitoneal (0.1 mg/kg) or subcutaneous (2 μg/ml) injection in PBS versus the IgG control antibody (Thermo Fisher Scientific, 02-6202).

### Immunofluorescence

Rats from each experimental group were terminally anaesthetised (Dolethal; intraperitoneal sodium pentobarbital, 200 mg/ml) and were transcardially perfused with PBS and subsequently with 4% paraformaldehyde (PFA; pH 7.4). Lumbar DRG and hindpaw plantar skin were extracted, submerged in PFA overnight and cryopreserved in 30% sucrose. Tissues were stored at −80°C until processing (Ved et al., 2018). Cryosections for DRG (8 µm thickness) and plantar skin (20 µm thickness) sections were acquired using a cryostat (Leica CM1860 UV) and collected onto SuperFrost Plus slides (VWR International). Slides were washed three times with PBS containing 0.2% Triton X-100 and then blocked with PBS containing 0.2% Triton X-100, 5% bovine serum albumin (BSA) and 10% foetal bovine serum (FBS) for 1 h at room temperature. Sections were incubated at 4°C for 72 h in blocking solution (5% BSA, 0.2% Triton X-100) containing the following primary antibodies and reagents: anti-CGRP (Sigma-Aldrich, C8198, 1:500), anti-CD45 (Abcam, ab10558, 1:400), anti-CD11b (Abcam, ab133357, 1:200), anti-NGF (Sigma-Aldrich, N8773, 1:100), guinea pig anti-NeuN (Synaptic Systems, 266 004, 1:100) and biotinylated IB_4_ (Sigma-Aldrich, L2140, 1:200). Sections were washed in PBS for 5 min three times. Secondary antibodies were subsequently applied to the tissue sections in PBS containing 0.2% Triton X-100 at room temperature for 2 h. CGRP immunoreactivity required an additional incubation step for 2 h at room temperature to incorporate anti-rabbit biotinylated IgG (The Jackson Laboratory, 711-067-003, 1:500), followed by incubation with streptavidin-conjugated Alexa Fluor 555 (Invitrogen, S32355, 1:1000). The secondary antibodies (1:1000) used were: Alexa Fluor 555-conjugated donkey anti-mouse (Abcam, ab150114), Alexa Fluor 555-conjugated donkey anti-rabbit (Abcam, ab15158) and Alexa Fluor 488-conjugated donkey anti-rabbit (Abcam, ab150073) IgGs. All antibodies have previously been validated by us (Da Vitoria Lobo et al., 2022; Hardowar et al., 2024) and performed alongside no-primary controls. Coverslips were mounted using VectaShield (Vector Laboratories, H1000) mounting medium. Sections were imaged using a Leica confocal microscope.

### Endothelial cell culture

HUVECs (PromoCell, C-12203) were cultured in endothelial growth supplement (Sigma-Aldrich, E2759) and plated in either six- or 24-well plates. For western blot analysis, 50,000 cells per well were seeded on six-well plates, and for immunofluorescence assays, 5000 cells per well were seeded in 24-well plate with ethanol-sterilised coverslips. Cells were left to grow until they reached 90% confluency and then treated with cisplatin (0, 1, 3 or 5 μg/ml) for 24 h. All reagents used were from Lonza.

Lumbar spinal cords were extracted from C57BL/6 mice. Isolated spinal cords were incubated in endothelial cell medium [consisting of M199 medium (Sigma-Aldrich), 60 mg/ml endothelial cell growth supplement (Sigma-Aldrich) and 50 μg/ml heparin (Sigma-Aldrich)] containing 1.25% collagenase (Sigma-Aldrich) for 30 min. The cell suspension was centrifuged (240 ***g***, 5 min) and the supernatant was removed. The cell pellet was resuspended, placed on top of a 15% BSA solution (Sigma-Aldrich) and centrifuged (1200 ***g***, 20 min). The subsequent cell pellet was resuspended in medium. Isolated spinal cord endothelial cells were plated on 1% gelatin coated six- or 24-well plates.

### Immunocytochemistry

Coverslips from 24-well plates were fixed with 1% PFA for 10 min, then washed three times with PBS containing 0.1% BSA, followed by one wash of PBS containing 0.1% Tween 20 and three more washes with PBS containing 0.1% BSA, and blocked using the blocking solution. Primary antibodies for VE-cadherin (Abcam, ab33168, 1:100) or ICAM1 (Santa Cruz Biotechnology, 1:200) were added and left overnight at 4°C. Three washes with PBS containing 0.1% BSA were performed after the primary antibody incubation. Secondary antibodies (Alexa Fluor 488- and 555-conjugated anti-rabbit IgGs) were added in a 1:500 dilution for 1 h at room temperature. Three more washes were performed, and the coverslips were carefully removed from the 24-well plate, placed onto SuperFrost slides (Sigma-Aldrich) and mounted with VectaShield mounting medium. Coverslips were sealed with nail polish. All images were taken using the LAS X software from Leica Microsystems for confocal microscopy (TCS SPE confocal microscope).

### Splenocyte adherence assay

Spleens were removed from adult C57BL/6 mice and were passed through a 40 μm strainer using RPMI 1640 medium (Invitrogen), penicillin-streptomycin (Sigma-Aldrich), 10% FBS (Invitrogen), L-glutamine (Invitrogen), sodium pyruvate solution (Sigma-Aldrich) and monothioglycerol (Sigma-Aldrich). Cells were incubated in Red Blood Cell Lysing Buffer (R7757, Sigma-Aldrich) prior to experimentation. Splenocytes were labelled with Vybrant Dil (Sigma-Aldrich, 468495) before incubation with cisplatin-treated HUVECs. Splenocytes in suspension were incubated at 37°C and 5% CO_2_ with 5 μl of Vybrant Dil solution per millilitre of suspension for 20 min. The medium was removed by centrifugation and the washing procedure was repeated twice. Splenocytes were incubated with cisplatin-treated HUVECs for 24 h in 24-well plates. The cells were then fixed with 1% PFA for 10 min and subsequently washed five times with PBS. Coverslips were removed from the plate, placed onto SuperFrost slides using Vectashield mounting medium and finally sealed with nail polish for confocal microscopy imaging.

### Flow cytometry of mouse splenocytes

Spleens were removed from adult C57BL/6 mice and passed through a 40 μm strainer using RPMI 1640 medium (Invitrogen), penicillin-streptomycin (Sigma-Aldrich), 10% FBS (Invitrogen), L-glutamine (Invitrogen), sodium pyruvate solution (Sigma-Aldrich) and monothioglycerol (Sigma-Aldrich). Cells were incubated in Red Blood Cell Lysing Buffer (R7757, Sigma-Aldrich) prior to cell culture. Splenocytes were treated with either vehicle or cisplatin (5mg/ml) for 24 h. Following this, the cells were fixed with 4% paraformaldehyde and permeabilised with 0.4% Triton X-100 in PBS. Cells were incubated in either conjugated antibodies CD11b-APC (1:100; BioLegend, San Diego, CA, USA) or F4/80-PE (1:100; Biolegend)] in combination with NGF antibody (goat anti NGF, Sigma-Aldrich, N8773) and left overnight. The cells were then washed in PBS, and secondary antibody anti-goat Alexa Fluor 488 was added (1:1000, Thermo Fisher Scientific, A-11055). Samples were analysed on a MoFlo analyser.

### Western blotting

Proteins from cisplatin-treated HUVECs were extracted using RIPA buffer (Thermo Fisher Scientific) with 1× protease inhibitor cocktail (Thermo Fisher Scientific, 78440). Protein lysates was equally loaded onto 10% Precast gels (Bio-Rad), with 50 μg protein per well. Membranes were blocked with TBS containing 1% BSA and 0.1% Tween 20 for 1 h at 4°C. Primary antibodies were incubated overnight at 4°C in a 1:200 dilution for ICAM1 (Santa Cruz Biotechnology, sc-7891), β-actin (Santa Cruz Biotechnology, sc-1616) and VE-cadherin (Abcam, ab33168, 1:100). Three washes with TBS containing 0.1% Tween 20 (TBST) were performed after the incubation. Secondary LI-COR antibodies were incubated in a 1:5000 dilution for 1 h at room temperature. Five final washes with TBST were performed, and membranes were then analysed with the LI-COR Odyssey imager.

### Splenocytes and NGF protein quantification

Protein lysate samples were extracted from mouse splenocytes. Splenocytes were plated into six-well plates for 24 h prior to cisplatin (5 µg/ml) treatment. Cells were lysed using RIPA buffer (Sigma-Aldrich) containing protease/phosphatase inhibitor cocktail (Cell Signaling Technology). Equal protein lysate concentrations (40 μg per well) were loaded on a 4-20% precast Mini-Protean gradient TGX gel (Bio-Rad). Proteins were separated by SDS-PAGE and transferred to PVDF membranes using a Trans-blot turbo transfer system (Bio-Rad). Membranes were incubated in 5% BSA in TBST for 1 h at room temperature. Primary antibodies were incubated overnight at 4°C [anti-NGF (goat, Sigma-Aldrich, N8773), CD45 (Abcam, ab10558, 1:400), β-actin (rabbit, Santa Cruz Biotechnology, sc-1616, 1:100)]. Membranes were then incubated in secondary antibodies [LI-COR donkey anti-rabbit, anti-goat and anti-mouse antibodies, 1:10,000] in TBST containing 1% BSA. Membranes were then washed three times with TBST and visualised on the LI-COR Odyssey Fc imager.

### Primary DRG sensory neuronal cell culture

Prior to dissection, 75 μl per well of poly-L-lysine was added to 96-well plates and incubated overnight. The wells were washed with PBS, left to dry, then 100 μl of 5 μg/ml laminin in PBS was added to each well. DRG were extracted from C57BL/6 mouse pups aged between P0 and P7 (five pups per culture) and collected in Ham's F-12 medium (Thermo Fisher Scientific), 3% BSA, penicillin/streptomycin and N-2 supplement (Thermo Fisher Scientific). Enzymatic digestion was performed using collagenase type IX (Sigma-Aldrich), with DRGs incubated for 2 h. This cell suspension was mechanically triturated using a 1 ml pipette tip to ensure the complete dissociation of the neurons, with 1 ml of the cell suspension added to each of two 15% BSA cushions (1 ml Ham's F12 medium and 1 ml 30% BSA) in 15 ml falcon tubes and centrifuged at 1200 ***g*** for 8 min. The supernatants containing the cell debris, BSA and medium were aspirated, and the cell pellets containing the neurons were resuspended in 1 ml of medium. DRG sensory neurons were seeded at a concentration of 2000 cells per well.

### DRG sensory neuronal calcium assay

*In vitro* DRG sensory neuronal intracellular calcium assay was performed as previously described (Bestall et al., 2018; Hulse et al., 2014). NGF (Alomone) and capsaicin (Sigma-Aldrich) were added at final concentrations of 1 nM and 1 μM, respectively, 24 h prior to imaging. For the cisplatin treatments with NGF and the NGF-neutralising antibody, all treatment groups were treated with 5 μg/ml cisplatin made up in supplemented F12 medium 1 week prior to imaging, with the medium replaced with fresh medium 24 h later (6 days prior to calcium assay). We used the Fluo-4 calcium indicator (Thermo Fisher Scientific), which has been previously used to investigate neuron calcium handling (Higham et al., 2024). DRG sensory neurons were loaded with 100 μl per well of Fluo-4 cell permeant in medium containing 5% pluronic acid and incubated for 1 h. Capsaicin-evoked activity was measured by the Infinite M Plex plate reader (Thermo Fisher Scientific). The fluorescence response per well was measured at 10 s intervals over a 200 s period. Prior to imaging, the plate reader was set to measure at 37°C to reflect physiological temperatures. The wavelength settings were selected based on the manufacturer’s specifications (488 nm excitation and 516 nm emission), with the gain set to 185. Baseline fluorescence readings were taken prior to the administration of capsaicin treatment. Baseline recordings were at t=0. Following this, capsaicin was added to induce intracellular calcium influx with all subsequent timepoint readings post t=0 expressed as a fold change over baseline to reflect capsaicin-induced responses.

### Gene expression analysis using RNA sequencing

Two to five lumbar DRGs were extracted and pooled from age-matched vehicle-treated and cisplatin-treated rats at P23, the timepoint at which CINP was induced and maintained. RNA sequencing was performed by Novogene. Messenger RNA was purified from total RNA using poly-T oligo-attached magnetic beads. After fragmentation, the first strand of cDNA was synthesised using random hexamer primers, followed by second-strand cDNA synthesis using either dUTP for directional library or dTTP for non-directional library. The non-directional library was ready after end repair, A-tailing, adapter ligation, size selection, amplification and purification. The directional library was ready after end repair, A-tailing, adapter ligation, size selection, USER enzyme digestion, amplification and purification. The library was checked with a Qubit fluorometer and real-time PCR for quantification and on a Bioanalyzer for size distribution detection. Quantified libraries were pooled and sequenced by Novogene using the Illumina NovaSeq PE150 platform, a paired-end sequencing technology with 150-bp read length. Raw reads were first cleared of adaptor sequences. At the same time, Q20, Q30 and GC content was calculated. All the downstream analyses were based on the clean data with high quality. Clean reads were mapped against the rat reference genome (ensemble_rattus_norvegicus_rnor_6_0_gca_000001895_4) using read aligner HISAT2 (v2.0.5). Mapped reads were assembled into transcripts or genes using StringTie software (v1.3.3b). Differential gene expression analysis was performed on raw counts using the R statistical package DESeq2 (v1.20.0). Genes with a >1.5-fold increase in expression [fold change (FC)≥1.5] and *P*<0.05 were considered significantly upregulated, and genes with a >1.5-fold decrease in expression (FC≤1.5) and *P*<0.05 were considered significantly downregulated. Non-coding genes were filtered out of the analyses. Gene enrichment pathway analysis was performed using STRING with the most abundant biological processes and molecular function highlighted, and a false discovery rate of <0.05 applied to adjusted *P*-values*.*

### Statistical analysis

All data are represented as mean±s.e.m. unless otherwise stated. Data were acquired and quantified using Microsoft Excel 2010, ImageJ (https://imagej.net/) (Schindelin et al., 2012; Schneider et al., 2012) and Graphpad Prism 8. Sample sizes were concluded via *a priori* sample size calculations. Raw Ca^2+^ response values obtained were collated and background fluorescence removed by subtracting the smallest measured fluorescence value from each value in the dataset. These new values were normalised by dividing by the fluorescence value obtained prior to capsaicin addition as a control, to determine the fold response over basal levels. For the capsaicin dose-response, a two-way ANOVA was used to determine the treatment effect over time at different concentrations. For all other datasets, the effects of different treatments on the Ca^2+^ neuronal response were compared by area under the curve analysis using two-tailed unpaired Student’s *t*-test, and one-way ANOVA tests with Bonferroni test were used depending on the data retrieved from each experiment. For immunoreactivity/immunohistochemistry studies, a minimum of four images per animal were acquired and the presented data are average of these values per animal. The immunoreactivity for CD45 was determined in the identified region of interest. Nociceptive behavioural assays were determined using one-way ANOVA with Bonferroni test. Immunohistological analysis used two-tailed unpaired *t*-tests or one-way ANOVA with Bonferroni test.

## Supplementary Material

10.1242/dmm.052062_sup1 Supplementary information
