# Supplementary material for: Early-life cisplatin exposure induces neuroinflammation and chemotherapy-induced neuropathic pain
Source: Dis Model Mech. 2024 Nov 27;17(11):dmm052062. doi: 10.1242/dmm.052062 (PMC11625889; doi:10.1242/dmm.052062)
Supplement: Supplementary information [file dmm-17-052062-s1.pdf]

A Molecular Function Pathway - Receptor ligand activity

Gene name	Log2FC		Gene name	Log2FC
Insl6	4.07146893		Cxcl11	1.55900492
Gcg	3.93421873		Tslp	1.47231809
Bmp8a	3.85542702		Gh1	1.43383025
Il19	3.75390534		Il17f	1.42443715
Thpo	3.64762055		Vip	1.41713437
Il12b	3.59650107		Ucn2	1.39499553
Tnfsf11	3.14808768		Ltb	1.34942631
Pyy	3.14135701		Ccl2	1.32515251
Ccl20	2.86512154		Ctsg	1.29408357
Ccl5	2.8190868		Wnt2	1.2372417
Tshb	2.36691956		Il24	1.16902647
Gdnf	2.34428717		Sfrp2	1.16384063
Ccl21	2.04494447		Tnfsf4	1.12601266
Gast	2.04084269		Cxcl13	1.08921817
Ntf4	2.01126939		Ccl3	1.08921817
Ccl1	2.00514869		Nppb	1.04477467
Mif	1.62980637		Cxcl9	1.01225919
Cxcl2	1.59969785		Ccl7	1.00888988
Colec10	1.56096296		Ngf	1.001284
Cxcl1	1.55900492			

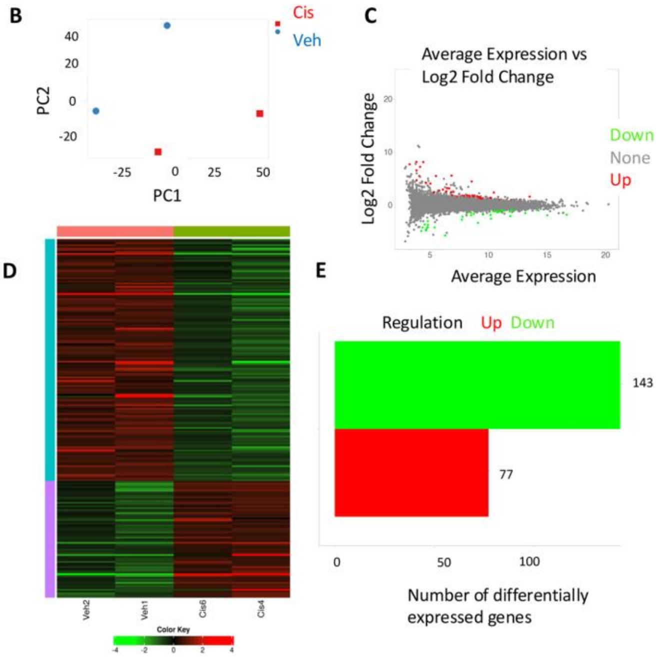

Fig. S1. Transcriptomic analysis of genes regulated by cisplatin in DRGs. Wister-Hans rats were treated with 0.3mg/kg cisplatin or saline (control) on days 14 and 16 postnatal. On P23 timepoint of pain development DRGs were isolated and mRNA extracted, and transcripts assessed using DESeq2 workflow. [A] Cisplatin induced a number of proinflammatory mediators including NGF in the DRG as indicated by the molecular function pathway analysis. [B] Shows Principal Component Analysis (PCA) plot for all genes, [C] MA scatter plot depicting all normalised genes with significantly upregulated genes shown in red and down regulated genes shown in blue. [D] Represents a heat map of all significantly expressed genes vehicle vs cisplatin treatment and [E] Is a bar chart detailing significantly upregulated and down regulated genes with protein coding genes listed below. Experimental n=2, Significantly expressed genes were determined using a fold change cut off ≥1.5 and P value of 0.05 with0.05 FDR (Benjamini-Hochberg).

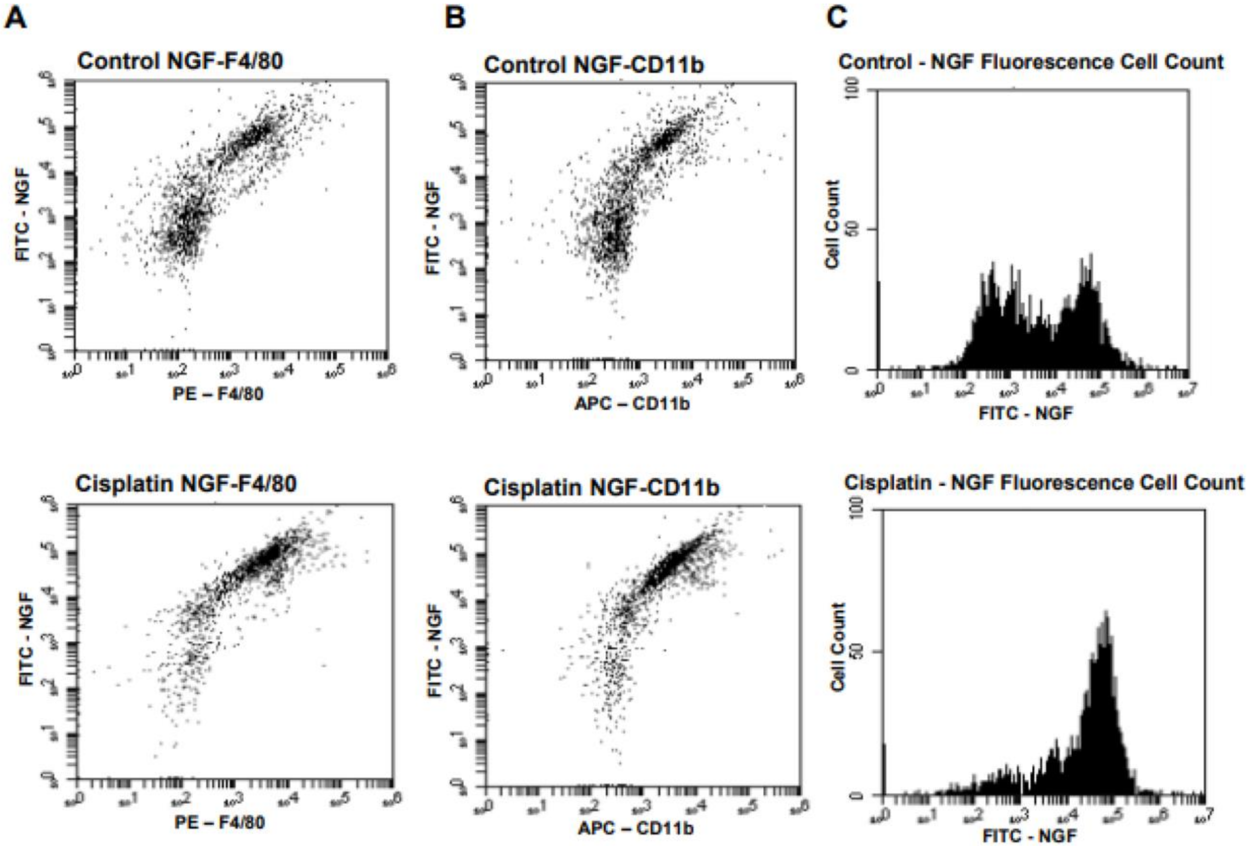

Fig. S2. Cisplatin induced NGF expression in macrophages. [A-C] Flow cytometry was used to measure expression of NGF in in F480 and CD11b positive immune cells following control (top) and cisplatin (bottom). Isolated mouse splenocytes treated with either vehicle or cisplatin for 24hrs led to increased NGF mean fluorescence intensity following cisplatin treatment (Unpaired t Test, *** p<0.001, n=5 per group).

Disease Models & Mechanisms • Supplementary information

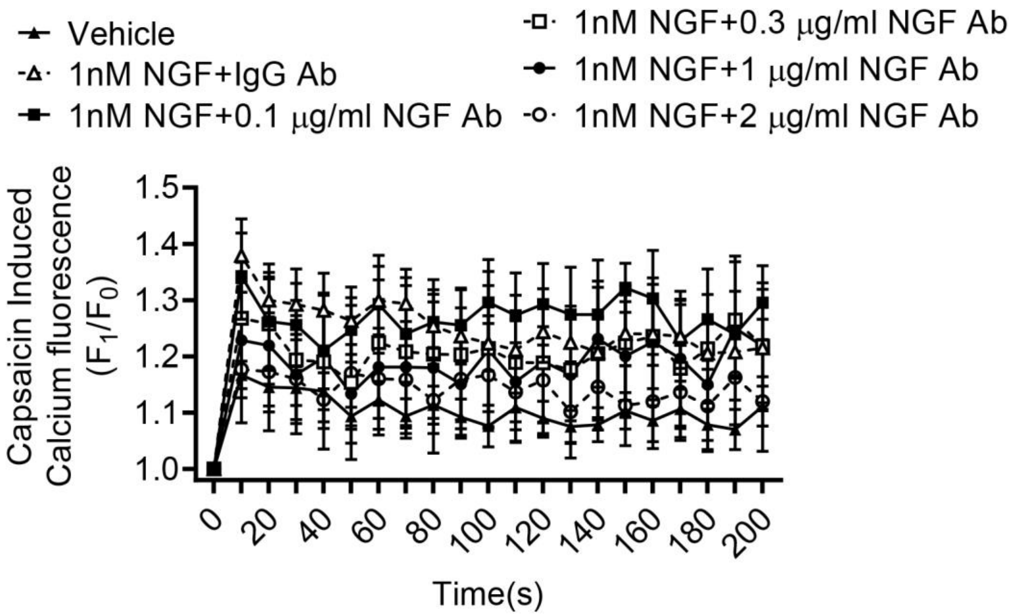

Fig. S3. Neutralising antibody suppression in NGF induced nociceptor senstisation and aberrant growth. Timelines of capsaicin induced intracellular calcium fluorescence of all concentrations of NGF Ab introduced in isolated DRG primary sensory neuronal cell cultures, NGF induced increase capsaicin mediated intracellular calcium influx, which was suppressed in a concentration dependent manner with increasing concentrations of NGF neutralising antibody (n=16).

Disease Models & Mechanisms • Supplementary information

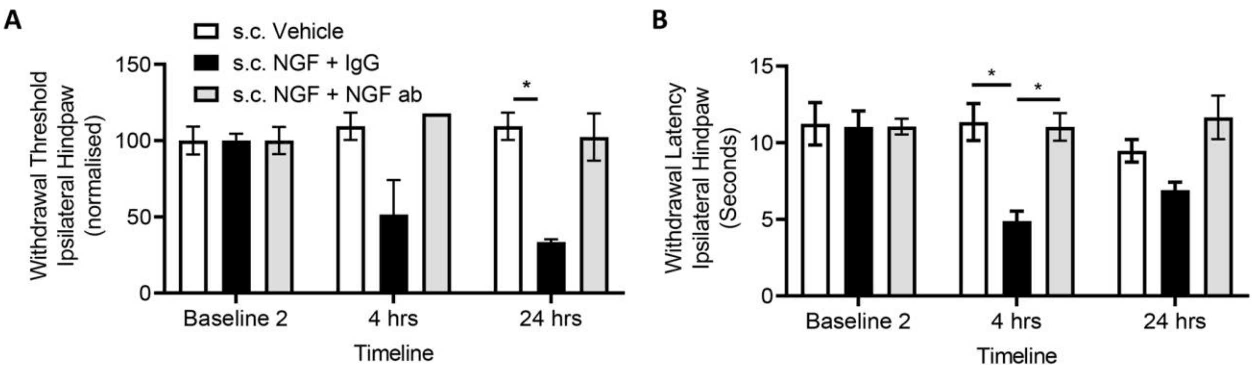

Fig. S4. NGF-induced mechanical and thermal hypersensitivity are prevented by inhibiting NGF/TrkA signalling. Wistar Hans rats were injected with either PBS (white), NGF, black) or NGF along with a NGF ab (grey). NGF was administered in all cases via intraplantar injection. NGF induced both mechanical [A] and thermal [B] hypersensitivity within 4 hours, with intraperitoneal injection of NGF ab preventing NGF induced pain. [C&D] Contralateral sensitization was observed following NGF administration with regards to mechanical hypersensitization. *p < 0.05, **p < 0.01 and ***p < 0.001, one-way ANOVA (Kruskal-Wallis test) compared to the NGF treated group. For experiment 1, n=6 for all groups; for experiment 2, n=6 for control and n=8 for both NGF and NGF+NGFab groups. Error bars are mean+SEM.

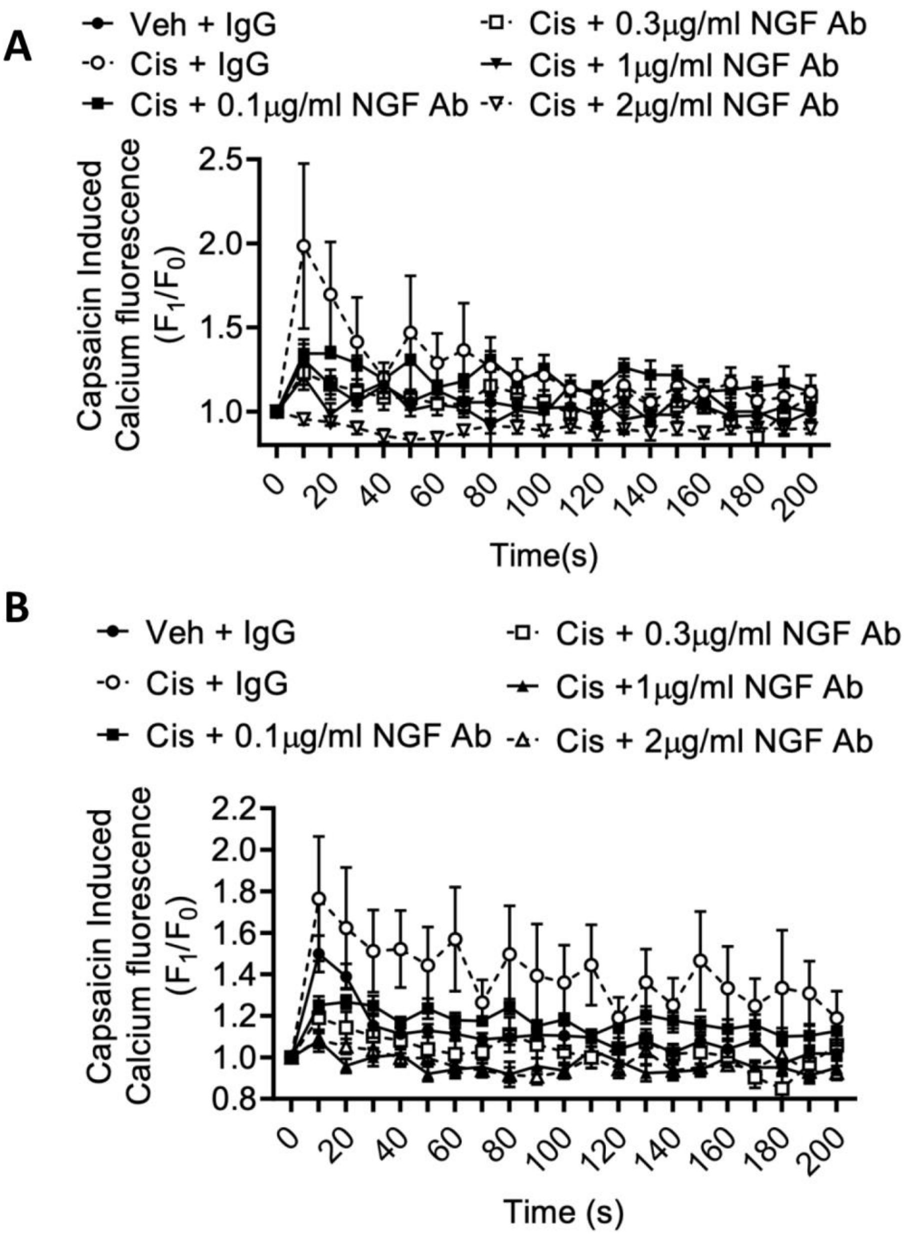

Fig. S5. Cisplatin induced proinflammatory mediated nociceptor sensitisation is NGF dependent [A]. Timelines of capsaicin induced intracellular calcium fluorescence of all concentrations of NGF Ab introduced in isolated DRG neurons following 24hr treatment of with 5µg/mL cisplatin 1 week prior to NGF treatment increased TRPV1 activity when compared to vehicle. NGF neutralising antibody diminished capsaicin induced TRPV1 mediated nociceptor activity. [B] Conditioned media from cisplatin treated mouse splenocytes led to increased capsaicin evoked DRG nociceptor activity compared vehicle treated conditioned media from mouse splenocytes, which was diminished with increasing concentrations NGF neutralising antibody.
